# Crop GraphRAG: pest and disease knowledge base Q&A system for sustainable crop protection

**DOI:** 10.3389/fpls.2025.1696872

**Published:** 2026-01-05

**Authors:** Hao Wu, Nengfu Xie, Xiaoli Wang, Jingchao Fan, Yonglei Li, Zhibo Meng

**Affiliations:** 1 Agricultural Information Institute, Chinese Academy of Agricultural Sciences, Beijing, China; 2 Key Laboratory of Agricultural Blockchain Application, Ministry of Agriculture and Rural Affairs, Beijing, China

**Keywords:** diseases and pests, knowledge graph, RAG, intelligent question answering system, crop protection

## Abstract

Intelligent prevention and control of crop diseases and pests is a critical link in safeguarding food security. However, agricultural practitioners often face fragmented information and low retrieval efficiency when seeking accurate, actionable knowledge. Furthermore, general-purpose large language models (LLMs) are prone to providing inaccurate or erroneous answers when applied to these specialized domains. To address these challenges, we assembled a large-scale corpus of knowledge on crop diseases and pests. Via entity and relation extraction, we constructed a multi-relational knowledge graph covering crops, diseases, pests, symptoms, and control measures. We subsequently designed Crop GraphRAG, a new framework that integrates knowledge graphs with retrieval-augmented generation (RAG). This system enables local knowledge-base question answering by retrieving adjacency subgraphs for relevant entities alongside summary-based passage retrieval. To evaluate performance, we curated a domain-specific test suite of question–answer pairs and conducted comparative and ablation experiments. Our experiments demonstrate that the Crop GraphRAG framework offers distinct advantages in answer accuracy and coverage compared to baselines. Crucially, the framework effectively suppresses hallucinated content, a common issue in generative models. These results verify the practical utility of the Crop GraphRAG framework for vertical-domain question answering. By mitigating the limitations of large language models in specialized agricultural contexts, this study provides a pragmatic tool for intelligent QA in the agricultural domain and advances the application of AI in crop protection.

## 1 Introduction

Agriculture is the foundation of the national economy; thus, promoting steady agricultural development and safeguarding food security are of paramount importance. Crop diseases and insect pests, as major factors that threaten food security and cause economic losses, have attracted increasing attention worldwide (Upadhyay et al., 2025). According to statistics from the Food and Agriculture Organization of the United Nations, approximately 20 percent to 40 percent of global crop production is lost each year to pests and diseases, which poses a serious threat to global economic development. China is the world’s largest producer of staple grains such as rice and wheat and continues to suffer recurrent impacts from these hazards; losses attributable to pests and diseases account for more than 65 percent of total losses each year (Wu et al., 2022). According to incomplete statistics, in 2019 the fall armyworm affected 26 provinces in China, with an impacted crop area reaching 112 million hectares (Wang et al., 2022). Therefore, effective prevention and control of pests and diseases are critical for both governmental authorities and farmers.

In the digital era, knowledge and information related to crop diseases and pests have increased rapidly and are distributed across heterogeneous sources such as scholarly articles, government reports, and news media, resulting in fragmentation and unclear semantic relationships (Vidya et al., 2025). Traditional methods of knowledge acquisition that rely on keyword-based retrieval often return many irrelevant results and perform poorly in deep semantic understanding and in the analysis of complex relationships (Yang et al., 2021). In recent years, a series of large language models represented by OpenAI’s ChatGPT 4 (Yao et al., 2024) have provided strong technical support for advances in natural language processing; however, these models still face significant challenges in specialized domains such as crop disease and pest management.

First, the model may exhibit hallucinations when answering questions. Because pretraining cannot ensure the suitability of its internal knowledge for specialized domains, and responses in the domain of crop diseases and pests require extensive specialized terminology, the model readily produces inaccurate answers and may further distort or modify the generated content. Second, general purpose large language models often lack traceability and interpretability when addressing questions in such professional contexts (Chen et al., 2024). Some researchers have employed supervised fine tuning (Liu et al., 2025) to endow models with these capabilities; however, whether through full parameter fine tuning or parameter efficient fine tuning (Ding et al., 2023), the timeliness of knowledge updates remains constrained by the training cutoff, and the problem of delayed knowledge refresh persists. Moreover, fine-tuned models frequently exhibit learning difficulties, underfitting, and parameter drift, manifested as low accuracy on the target task or poor generalization to unseen data (Wang et al., 2025).

Retrieval-augmented generation (RAG) (Arslan et al., 2024) enhances large language model responses by retrieving relevant information from an external knowledge base. Although RAG can circumvent retraining the base model, it typically requires converting large volumes of text into vectors and performing keyword-based retrieval, and its retrieval efficiency and holistic understanding of the corpus remain limited. Recently proposed GraphRAG (Ngangmeni and Rawat, 2025), employs a knowledge graph as the underlying knowledge base. A knowledge graph (Peng et al., 2023) is a knowledge representation framework that organizes unstructured data into a semantic network composed of entities, relations, and attributes, thereby making inter-data relationships explicit. This structure substantially improves retrieval efficiency and can effectively mitigate hallucinations when addressing specialized domain questions.

In this study, we constructed a domain-specific knowledge base for crop pests and diseases. It spans 28 major crop categories—including cereal grains (Poaceae), tuber crops, fiber crops, solanaceous crops, cucurbits, legumes, oil crops, sugar crops, and tobacco—covering 96 representative crops (e.g., wheat, rice, maize, and cotton) and more than 3,100 curated pest–disease records. The number of records per category is relatively balanced, showing only modest variation around the mean (≈111.4 records per category). Each record primarily includes the pest/disease name, symptoms of damage, occurrence factors, and control methods. We further propose a novel Crop GraphRAG framework that uses domain-specific prompting for entity and relation extraction, eliciting structured outputs from large language models. During graph construction, the Leiden community detection algorithm (Park et al., 2024) was selected rather than Louvain because it guarantees internally well-connected communities, achieves higher partition quality and stability, and exhibits faster convergence with better scalability on large networks—properties required by our large, semantically weighted graph. Communities were then used to aggregate semantically related text chunks and to establish a multi-level hierarchy, and concise community-level summaries were generated for each entity and relation. During retrieval, summaries are traversed from lower to higher levels to provide multi-granular context, which enhances the model’s understanding of the source texts and enables more accurate answer generation (Wan et al., 2025).

## 2 Method

**Figure 1:**
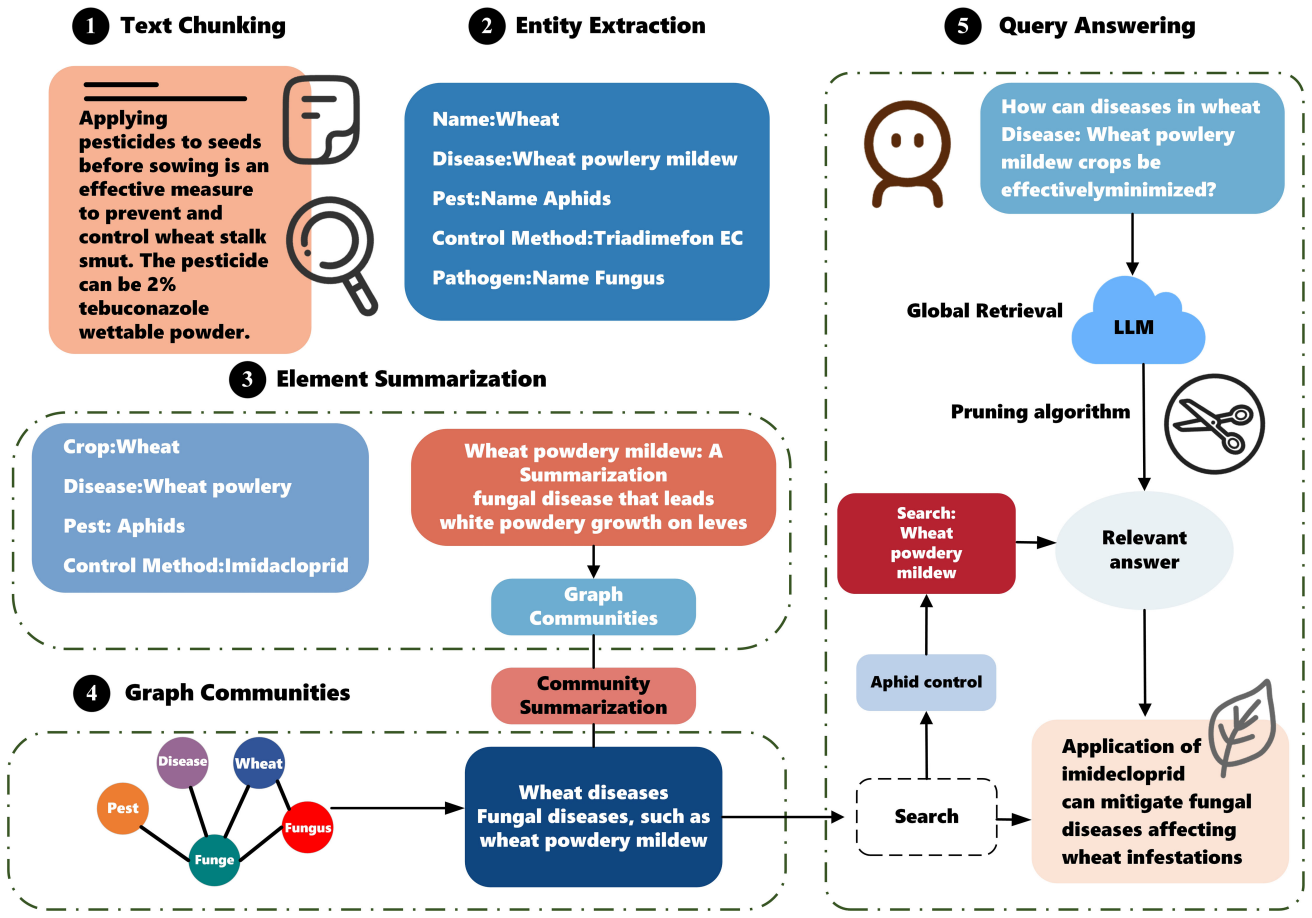
Crop GraphRAG framework diagram.

In this paper, we design and implement the Crop GraphRAG framework and use it to construct a knowledge base for crop diseases and pests. The detailed workflow of Crop GraphRAG is shown in **Figure 1** and is divided into five components. The main steps are as follows: 1) denoising and cleaning the collected unstructured data on crop diseases and pests (Yan et al., 2025); 2) extracting entities and relations; 3) constructing the knowledge graph; 4) generating community summaries; and 5) enabling knowledge graph retrieval and user–system question answering through an integrated architecture that combines file storage, a vector database, and local indices.

### 2.1 Construction of the knowledge graph for crop diseases and pests

Building the knowledge graph for crop diseases and pests is the first step of our framework. We describe the process from data preprocessing to the formation of the knowledge graph. The procedure comprises text segmentation, element extraction, element summarization, graph community detection, and community summarization.

#### 2.1.1 Text segmentation

The primary data sources used in this study fall into four categories: research articles, reviews, and technical bulletins published in agricultural journals; national-level specifications and guidelines for pest and disease control (e.g., documents issued by the Ministry of Agriculture and Rural Affairs and the National Agro-Tech Extension and Service Center), as well as technical documents released by plant-protection agencies across multiple provinces and municipalities; publicly available key information from pesticide registrations and labels; and expert-reviewed entries from specialized agricultural websites. We convert these unstructured materials into structured comma-separated value (CSV) files through data cleaning. The procedure removes noise, redundancy, and invalid content and corrects nonstandard symbols (Mumuni and Mumuni, 2025). The resulting CSV files contain two columns: id and text, which conforms to the design of the Crop GraphRAG framework.

After completing data preprocessing, the text was first segmented into chunks suitable for large language models. The chunk length was fixed at S=1200 tokens to align with the context window of the embedding model and to provide sufficient margin for batched processing; this setting also preserves semantic coherence, preventing topic dispersion when the granularity is too large and avoiding loss of contextual dependencies when it is too small. Adjacent chunks share an overlap of O=200 tokens to ensure that entities and relations spanning chunk boundaries are retained across multiple contexts. Based on analysis of the corpus, the average sentence length is 60–80 characters; therefore, an overlap of 200 tokens typically covers 2–3 complete sentences, substantially increasing the likelihood that the model captures complete semantic units. Let T denote the length of the original text and S the chunk size; the number of chunks N can then be computed as follows Equation 1.

(1) $$N=\left\lfloor \frac{T-O}{S-O}\right\rfloor$$

Here, ⌊·⌋ denotes the floor operator, which ensures that the number of chunks is an integer. This design retains more contextual information during segmentation and thereby preserves semantic coherence. Furthermore, the texts are grouped by ID, with the collection for group *i* denoted as $\left\{x_{i1},x_{i2},\ldots ,x_{im}\right\}$; the corresponding concatenation formula is Equation 2.

(2) $${\text{T}}_{i}=\text{Concat}\left({\text{x}}_{i1},{\text{x}}_{i2},\ldots ,{\text{x}}_{im}\right),\text{ where }\forall {\text{x}}_{ij}\in {\text{id}}_{i}$$

Correspondingly, the number of chunks for group *i* can be expressed as Equation 3.

(3) $$N_{i}=\left\lfloor \frac{\left|T_{i}\right|-O}{S-O}\right\rfloor$$

**Figure 2:**
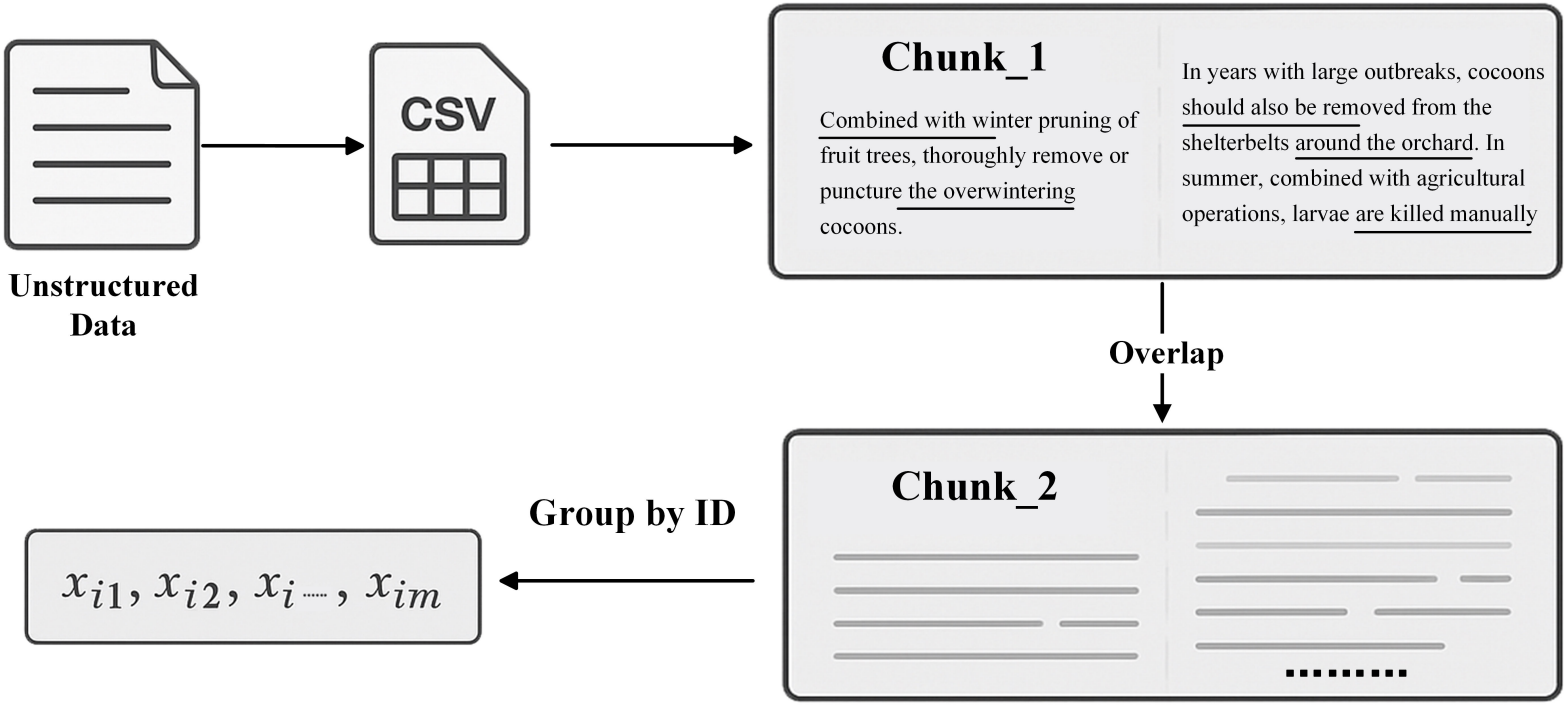
Text segmentation workflow. The figure illustrates the chunking process that converts unstructured agricultural text into structured knowledge units, thereby establishing high quality semantic units that support knowledge indexing and question answering in crop GraphRAG.

This processing strategy ensures that, for a given ID, all textual content associated with that ID is treated as a single unit during segmentation, as illustrated in **Figure 2** It prevents logical discontinuities and enhances the efficiency of subsequent semantic reasoning by the large language model.

#### 2.1.2 Element extraction

After segmenting the structured data, we design a prompt engineering scheme for a large language model and employ the model to extract textual elements, that is, to derive structured elements from each chunk. The extracted elements are organized into two primary categories: entities and relations (Harnoune et al., 2021). In this study, entities are defined as the core objects in the domain of crop diseases and pests, as summarized in **Table 1**.

**Table 1:** Definition of entity types.

Entity type	Examples
Crops	Rice; wheat; maize
Diseases	Rice blast; wheat stem smut; northern corn leaf blight
Insect pests	Rice stem borer; wheat thrips; millet darkling beetle
Symptoms	Root rot; hollow stem
Control measures	Biopesticides; physical control
Pathogen	Rice blast fungus

We define semantic links between entities as relations. Typical examples include pest harms crop, disease manifests as symptom, and pathogen causes disease.

Let each text chunk be $T_{i}$. After processing these chunks with a large language model, we obtain a structured set of triples $K_{i}$, given by Equation 4.

(4) $$K_{i}=\left\{\left(e_{s},r,e_{o}\right)|e_{s}\in ℰ,r\in ℛ,e_{o}\in ℰ\right\}$$

Here, ℰ denotes the set of entities and ℛ denotes the set of relations. Each triple $\left(e_{s},r,e_{o}\right)$ represents a semantic fact, indicating that relation r holds between the head entity $e_{s}$ and the tail entity $e_{o}$. The correspondence between the prompt engineering used in this study and the resulting triple set can be expressed as $\text{Prompt}\left(T_{i}\right)\to K_{i}$. The prompt comprises domain-specific keywords, reference question–answer templates, and exemplars of entity–relation mappings, which together provide strong reasoning capabilities for identifying entities and relations in this domain (Liu et al., 2023). We design the Extraction Prompt to be the following:

Given a text document and a list of entity types related to crop pest and disease activities, identify all entities of specified types in the text and the relationships between these entities.

1. Identify all entities. For each identified entity, extract the following information…
2. From the entities identified in Step 1, identify all *explicitly related* (source entity, target entity) pairs…
3. Return a single list of all entities and relationships identified in steps 1 and 2…
4. When complete, output {completion_delimiter}.

#### 2.1.3 Element summarization

After extracting entities and relations, individual entities or relations are often mentioned multiple times across the corpus, with their descriptions dispersed among different text chunks. To unify these descriptions and facilitate subsequent retrieval and question answering, we design a summarization module that receives the identifiers of entities or relations together with lists of source descriptions. Using entity linking, the module aggregates and merges these descriptions to the same entity. The resulting summary set D is defined as Equation 5.

(5) $$D={\left\{\left(id_{j},\left\{d_{j1},d_{j2},\ldots ,d_{jn_{j}}\right\}\right)\right\}}_{j=1}^{M}$$

Where $id_{j}$ denotes the identifier of the j−th entity or relation; $\left\{d_{j1},d_{j2},\ldots ,d_{jn_{j}}\right\}$ is the collection of descriptions associated with $id_{j}$ across all text chunks, and M is the total number of identifiers for entities and relations.

We further introduce a token upper bound τ to ensure that the input to the large language model does not exceed its context window. Excess tokens are truncated according to Equation 6.

(6) $${ℐ}_{j}=\text{Truncate}\left(\text{Concat}\left(d_{j1},d_{j2},\ldots ,d_{jn_{j}}\right),\text{τ}\right)$$

Where $\text{Concat}\left(d_{j1},d_{j2},\ldots ,d_{jn_{j}}\right)$concatenates, in order, the description fragments $\left\{d_{j1},d_{j2},\ldots ,d_{jn_{j}}\right\}$ that belong to the same entity or relation into a single text sequence. The large language model then applies the prompt engineering template to fill in the relevant entity and relation fields, producing for each entity a concise and comprehensive summary. These summaries provide detailed descriptions for nodes in the knowledge graph and support high quality retrieval and question answering.

#### 2.1.4 Graph communities

Using the methods described above, we obtain a knowledge graph composed of nodes and edges, where nodes correspond to entities and edges correspond to relations (Chandak et al., 2023). The overall structure can be formalized as a weighted undirected graph: G=(V,ℰ,w). Here, V denotes the set of entity nodes, ℰ denotes the set of relation edges, and $w:ℰ\to R^{+}$ is the edge-weight function, which reflects quantities such as relation occurrence frequency and semantic similarity.

However, the large volume of data poses substantial challenges for subsequent understanding and retrieval. Therefore, we adopt the Leiden algorithm to partition the entire graph. The Leiden algorithm is a community detection method that identifies and segments tightly connected groups of nodes within the knowledge graph (Park et al., 2024). The modularity used by the algorithm is defined as Equation 7.

(7) $$Q=\frac{1}{2m}\sum_{i,j}\left[A_{ij}-\frac{k_{i}k_{j}}{2m}\right]\text{δ}\left(c_{i},c_{j}\right)$$

In this expression, $A_{ij}$ indicates whether an edge exists between nodes i and j; $k_{i}$ denotes the degree of node i, that is, the sum of the weights of all edges incident to i; m is the total edge weight in the graph, $m=\frac{1}{2}\sum_{i,j}A_{ij}$; $c_{i}$ is the community label of node i; $\delta \left(c_{i},c_{j}\right)$ is the Kronecker delta (Jagtap et al., 2022), which equals 1 when $c_{i}=c_{j}$ and 0 otherwise. Accordingly, larger values of the modularity Q indicate more pronounced community structure. When Q is approximately 0, the graph structure is close to random; when Q>0, the community structure is discernible, and values above 0.3 are commonly regarded as meaningful, Negative values of Q imply that the partition performs worse than random and is therefore not meaningful. The Leiden algorithm improves upon the Louvain algorithm by addressing its instability and producing better connected, more reliable communities; its objective is to identify the partition that maximizes Q (Anuar et al., 2021).

By partitioning the knowledge graph, a hierarchical community structure is obtained. The community partition is expressed as Equation 8.

(8) $$C=\left\{C_{1},C_{2},\ldots ,C_{K}\right\},\;C_{k}\subseteq V,\;V=C_{1}\cup C_{2}\cup \cdots \cup C_{K}$$

A community is a set of nodes that are densely connected internally and sparsely connected externally. Each community $C_{k}$ contains a group of semantically closely related entity nodes. In the context of this study, a community corresponds to topics such as “major diseases affecting rice” or “insecticides suitable for fruit trees”.

#### 2.1.5 Community summarization

**Figure 3:**
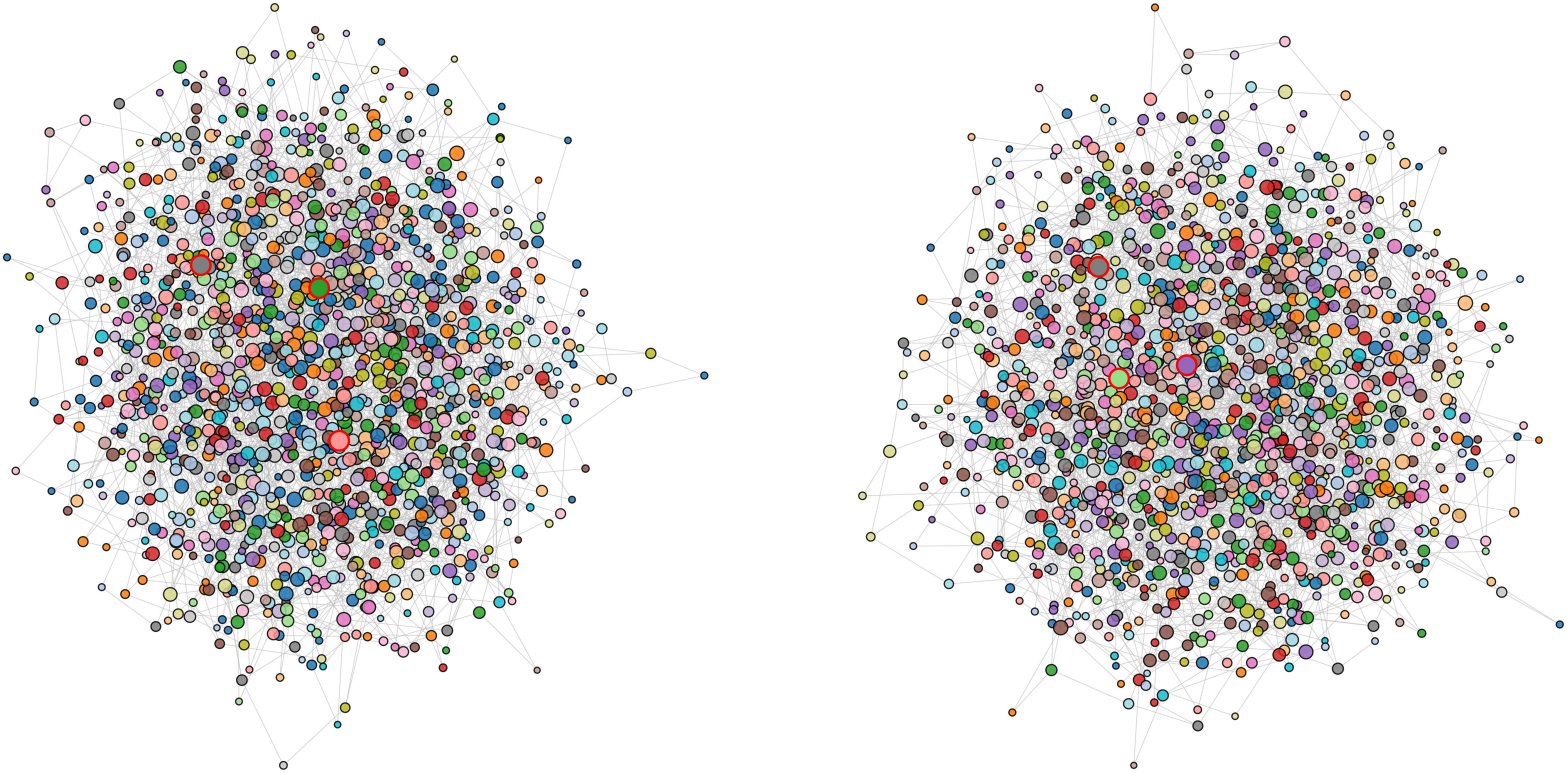
Comparison of leaf and higher-level communities. The figure presents the community partitioning and hierarchical organization derived from the knowledge graph of crop diseases and pests. The left panel shows the original community partition, where each color denotes a distinct semantic community. The right panel displays the aggregated higher level community structure, highlighting intercommunity connections and semantic aggregation. Each node represents an entity (for example, a disease or pest, or a crop type), and each edge represents a semantic relation between entities.

After partitioning the knowledge graph into communities, we generate an easily interpretable summary for each community, referred to as a community summary (Jin et al., 2021), to support subsequent querying and retrieval. These summaries constitute the top-level view of the Crop GraphRAG index and are a key component for enabling global question answering. Communities are further categorized into leaf communities and higher-level communities, as illustrated in **Figure 3**.

For each community, Crop GraphRAG aggregates evidence from both the source texts and the graph structure. From the textual side, it collects the post-segmentation text chunks together with the previously constructed element summaries. From the graph side, it gathers all entities, their attributes, and inter-entity relations. These inputs are then packaged and provided to a large language model, which leverages both textual and graph context under a specified prompt to produce a community summary, as formulated in Equation 9.

(9) $$S_{k}=\text{LLM}\left({\text{Prompt}}_{\text{leaf}}\left(T_{k},G_{k}\right)\right)$$

Where $T_{k}$ denotes the collection comprising the text chunks associated with the community’s entities and their element summaries, $G_{k}$ denotes the graph-structural information within the community, ${\text{Prompt}}_{leaf}$ is the prompt template for community summarization, and LLM(·) is the summary generation function. Each community summary covers the core theme of the community, the principal entities, the relations among those entities, and other salient information. This fine-grained unit is termed a leaf community, and the resulting outputs are referred to as leaf-community summaries (Zhang et al., 2021).

For the entire knowledge graph, we further construct a higher-level graph view. Building on the leaf communities, we merge them into an upper layer community $C_{i}^{\left(ℋ\right)}=\left\{C_{i1},C_{i2},\ldots ,C_{in_{i}}\right\}$. The corresponding summary $S_{i}^{\left(H\right)}$ is defined as Equation 10.

(10) $$S_{i}^{\left(H\right)}=\text{LLM}\left({\text{Prompt}}_{high}\left(\left\{S_{i1},S_{i2},\ldots ,S_{in_{i}}\right\}\right)\right)$$

Where $\left\{S_{i1},\ldots ,S_{in_{i}}\right\}$ denotes the merged set of leaf-community summaries. In constructing this set, neither the element summaries nor the graph-structural information described above are used; instead, the leaf community summaries are provided to the large language model to produce the aggregated summary for the upper layer community, denoted $S_{i}^{\left(H\right)}$. Finally, the summaries $S_{i}^{\left(H\right)}$ are combined hierarchically to yield the higher-level community summary $S^{\left(\text{Top}\right)}$, the corresponding Equation 11 is given as follows.

(11) $$S^{\left(Top\right)}=\text{LLM}\left({\text{Prompt}}_{root}\left(\left\{S_{1}^{\left(H\right)},S_{2}^{\left(H\right)},\ldots \right\}\right)\right)$$

By adopting this hierarchical approach, computational complexity is reduced, and a macro-level synopsis of the entire knowledge graph is obtained, which in turn significantly accelerates retrieval. At this stage, we have completed the construction of the knowledge graph, the community reports, and the indexing pipeline. The final outputs consist of six index files: the entity table, the relation table, the raw units, the text-chunk units, the community metadata, and the community reports.

### 2.2 Query and question answering

In this module, we design two retrieval strategies: local query answering and global query answering. Compared with conventional RAG retrieval, our framework innovatively performs graph-based retrieval within the knowledge graph and leverages community summaries. This design enables more accurate and comprehensive responses to domain-specific questions on crop diseases and pests.

#### 2.2.1 Local query

Local query refers to an entity-centric retrieval and reasoning strategy. When a user poses a question about a specific entity, the system searches the constructed knowledge graph to locate the corresponding entity node with precision. It then expands outward along the edges incident to that node, gathering entities and relations that are directly relevant to the question, together with associated source text fragments. The retrieved evidence is provided as contextual input to the large language model, which uses these prompts to generate answers with higher accuracy and factual reliability.

Local querying is subject to constraints. If the entity referenced in a user question is not clearly specified, imprecise retrieval can lead to inaccurate answers. Consequently, local querying is particularly suitable for factual and narrowly scoped questions. Examples include identifying the target pests of a specific pesticide (“Which pests is imidacloprid primarily used to control?”) and describing the characteristic symptoms of a specific disease (“What are the typical field symptoms of wheat powdery mildew?”).

During local querying, the system loads the index files generated during knowledge graph construction. Given a user question q, an encoder maps it to a query vector $v_{q}=\text{Encoder}\left(q\right)$, where $v_{q}\in R^{d}$denotes a dense *d*-dimensional representation (Zhao et al., 2024). Using the vector indices built for entities and relations, we denote the entity set by $ℰ=\left\{e_{1},e_{2},\ldots ,e_{n}\right\}$ and their embeddings by $V_{ℰ}=\left\{v_{e_{1}},v_{e_{2}},\ldots ,v_{e_{n}}\right\}$, Cosine similarity is then computed to identify the entity most similar to the query vector, yielding the most relevant entity $e^{*}$ (Steck et al., 2024), compared with Euclidean distance and the raw dot product, cosine similarity—owing to its scale invariance, robustness, and alignment with embedding-space geometry—better satisfies the production requirements of a crop pest and disease knowledge-based question answering system, as shown in Equation 12.

(12) $$e^{*}=\arg\underset{e_{i}\in ℰ}{\max}\cos\left(v_{q},v_{e_{i}}\right)$$

Here, $v_{e_{i}}$ denotes the embedding of the i−thentity in the knowledge graph, and $\cos\left(v_{q},v_{e_{i}}\right)$ is the cosine similarity between the query vector and the entity vector. In addition, by matching entities in the graph and centering on the most relevant entity $e^{*}$, the system constructs the one−hop adjacency subgraph $G_{e^{*}}^{\left(1\right)}$ within the knowledge graph G, defined as Equation 13.

(13) $$G_{e^{*}}^{\left(1\right)}=\left\{\left(e^{*},r_{j},e_{j}\right)\in G|\text{dist}\left(e^{*},e_{j}\right)=1\right\}$$

Concurrently, context fragments associated with the entity $e^{*}$are extracted from the source texts and the community summaries: $T_{e^{*}}=\text{TextChunks}\left(e^{*}\right)\cup \text{SummaryBlocks}\left(e^{*}\right)$;After retrieval, the system consolidates the extracted corresponding entity and all information associated with it into a structured data table ${\text{Table}}_{e^{*}}$, defined as Equation 14.

(14) $${\text{Table}}_{e^{*}}=\text{BuildTable}\left(e^{*},G_{e^{*}}^{\left(1\right)},T_{e^{*}}\right)$$

Finally, the data table is populated into the domain-specific prompt template for crop disease and pest management and supplied to the large language model as contextual input. The model then generates the precise final answer *a* according to the template. We formalize the local query pipeline as Equation 15.

(15) $$a={ℱ}_{\text{local}}\left(q\right)=\text{LLM}\left({\text{Prompt}}_{\text{local}}\left(\text{BuildTable}\left(e^{*},G_{e^{*}}^{\left(1\right)},T_{e^{*}}\right)\right)\right)$$

#### 2.2.2 Global query

Global querying relies on community summaries within the knowledge graph. When a user poses a more abstract question, the system does not attempt to match each individual entity. Instead, it exploits the hierarchical community structure of the knowledge graph and applies a pruning algorithm (Chen et al., 2021) to select the communities relevant to the query. The corresponding community summaries are then processed in parallel, and the results are aggregated to produce a higher-level, synthesized answer.

Compared with local querying, global querying imposes no such constraints. It can accommodate questions targeting specific entities as well as tasks such as topic synthesis and trend analysis. For example, “Summarize the principal disease and pest threats to maize in North China.”

At the onset of global querying, the system performs dynamic community selection based on the indices for communities and their summaries. Starting from the top level of the knowledge graph, it evaluates each community at the current layer by applying a specialized assessment prompt to the large language model, which judges the relevance between the summary of community $C_{i}$ and the user query q and returns a score from 0 to 5, defined as Equation 16.

(16) $$\text{Score}\left({\text{C}}_{i},\text{q}\right)=\text{LLM}\left({\text{Prompt}}_{score}\left(\text{Summ}\left({\text{C}}_{i}\right),\text{q}\right)\right)$$

Here, ${\text{Prompt}}_{score}$ denotes the specialized prompt that guides the large language model to make the relevance judgment, and $\text{Score}\left(C_{i},q\right)\in \left[0,5\right]$ is the discrete relevance score returned by the model. After analyzing the processed knowledge base on crop pests and diseases, we applied a pruning algorithm to distinguish communities and set the decision threshold to θ=3. This choice attains 100% recall of core-relevant communities (scores 3–5) while filtering 76% of weakly related or irrelevant content (scores 0–2). It reduces computational cost, accommodates the high-certainty characteristics of the domain, and conforms to the expected-risk minimization principle in Bayesian decision theory (McNamara and Chen, 2022), yielding a balanced operating point. For each community $C_{i}$ pruning is performed according to Equation 17.

(17) $$\{\;\begin{array}{c} \text{Keep}\left(C_{i}\right)=\;\text{True},\;\text{ if}\;\;\text{Score}\left(C_{i},\;q\right)\;>\;\theta \\ \text{Keep}\left(C_{i}\right)=\text{False},\;\text{ otherwise} \end{array}$$

If a community is judged highly relevant, its child communities are added to the next scoring queue and the search proceeds downward. Conversely, if a community is judged irrelevant, that community and all its descendants are pruned and no further exploration is performed, defined as Equation 18.

(18) $$\forall C_{j}\in \text{Descendants}\left(C_{i}\right),\text{ Prune}\left(C_{j}\right)=\text{True}$$

Using a top-down traversal, we obtain community-level summaries that are highly relevant to the user’s query. These summaries are supplied as contextual input to a large language model (LLM), which—guided by predefined prompts—induces a set of partial answers. The system then aggregates these partial answers into a final prompt that instructs the model to integrate, refine, and synthesize the fragmented evidence into a single response.

To handle contradictions or mutually exclusive statements across sources, we introduce a consistency-evaluation and conflict-resolution module. It applies weighted consistency optimization and retrieval re-ranking to prioritize globally consistent conclusions supported by high-weight evidence, while iteratively re-retrieving and rewriting low-consistency fragments (Li et al., 2025). A comprehensive citation system and verification labels are maintained to support targeted human-in-the-loop intervention when necessary.

## 3 Experiment

To systematically evaluate our domain-specific knowledge base for crop diseases and pests and to assess the effectiveness of the proposed system, we design multiple experimental protocols in this chapter. These experiments are intended to address the following questions:

1. When addressing domain-specific questions, does the local querying method in the Crop GraphRAG framework provide advantages in accuracy and relevance compared with traditional retrieval methods?
2. For questions that require analysis at the macro level, can the global querying method in the Crop GraphRAG framework produce deeper answers than alternative approaches?
3. How does the framework perform in practical application scenarios?

Accordingly, we design multiple experimental protocols to investigate these questions, evaluate the system’s concrete performance, and identify its limitations through comparative testing.

### 3.1 Dataset

We construct a domain dataset for crop diseases and pests. The sources include research articles from core Chinese agricultural journals, official guidelines for pest and disease prevention and control issued by the National Agro-Tech Extension and Service Center and provincial or municipal plant protection stations, and data acquired from specialized agricultural websites such as the Chinese Crop Disease and Pest Knowledge Base. We compiled more than 3,000 Chinese documents totaling approximately 15 million characters; after cleaning, deduplication, and normalization, the curated corpus contains about 6 million characters.

**Figure 4:**
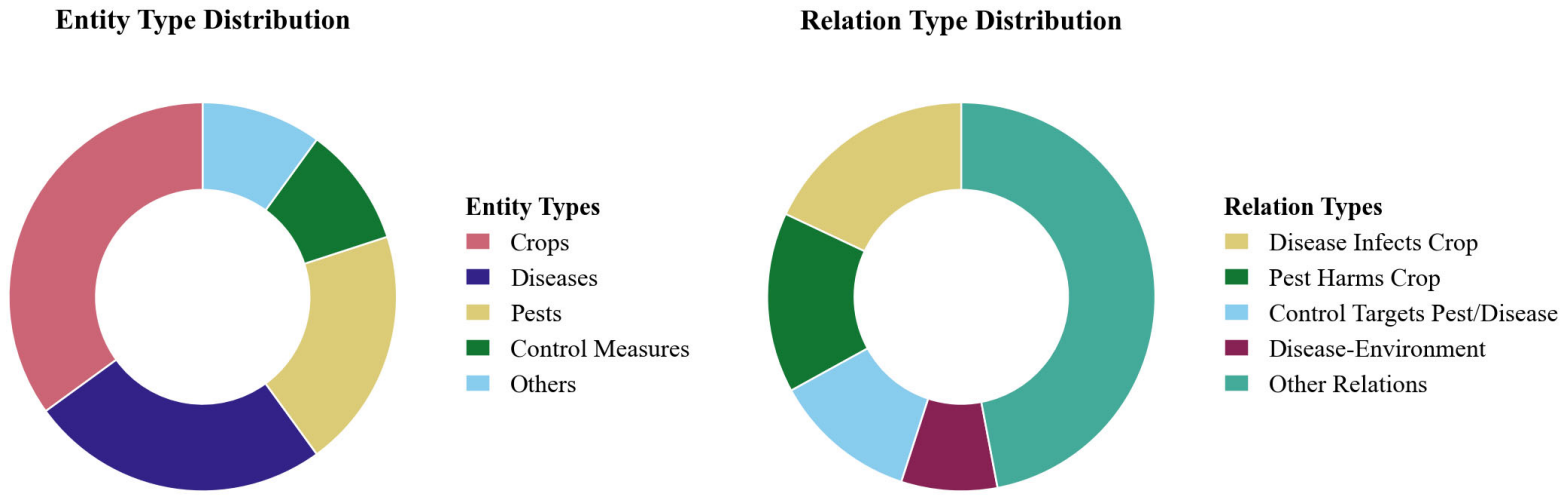
The detailed distribution of types.

Using this corpus, we constructed a domain-specific knowledge graph comprising more than 20,000 entities and approximately 60,000 semantic relations that capture interconnections among crops, pests, diseases, and environmental factors. Entities were grouped into five categories: crops (approximately 35%), diseases (25%), pests (20%), control measures (10%), and auxiliary entities such as environmental factors and pesticides (10%). In total, about 40 relation types were defined; the most frequent were “disease infects crop” (≈18%), “pest damages crop” (≈15%), “control measure targets pest or disease” (≈12%), and “disease associated with environmental factor” (≈8%), reflecting interaction patterns observed in agricultural practice. The graph covers more than 30 major crop species and over 100 common pest and disease types, supporting concise semantic representation and multi-dimensional reasoning. The detailed distribution of types is shown in **Figure 4**.

To evaluate the performance of our system’s different retrieval strategies in the crop disease and pest domain, we constructed an evaluation dataset comprising more than two hundred domain-specific questions. These questions were jointly authored by graduate students and experts in agriculture to ensure professional quality and authenticity.

We categorize the questions into two types: factual questions and analytical questions. Factual questions target specific entities and are used to assess the performance of local querying, whereas analytical questions require synthesis, generalization, or address broader topics. Representative examples are provided in **Table 2**.

**Table 2:** Examples of evaluation questions.

Category	Example questions
Factual question set	“Which triazole fungicides are commonly used to control wheat powdery mildew?” “What are the morphological characteristics of the adult rice leaffolder?”
Analytical question set	“Compare and analyze the advantages and disadvantages of biopesticides versus chemical pesticides in the control of tomato gray mold.” “Summarize the major insect pests and integrated management strategies for maize in the mid-to-late growth stages.”

### 3.2 Baselines

We compare the Crop GraphRAG framework with several models and conduct ablation studies. Traditional retrieval (BM25+LLM): the query is tokenized and converted to keywords; an inverted index with BM25 (Wang et al., 2021) is used for term matching; the retrieved passages are concatenated with the question and sent to an LLM. This serves as a reference upper bound for pure keyword retrieval. Naive RAG: text-only vector retrieval without the knowledge graph or community summaries; chunked texts are embedded to build a FAISS (Filipovska et al., 2025) index, evidence is retrieved by similarity, and an LLM generates the answer. GraphRAG-wo-Community (without community summarization): the community module is removed; the system relies on entity-node retrieval and aggregation of neighborhood evidence. GraphRAG-wo-KnowledgeGraph (without the knowledge graph): the graph structure is removed; the system uses only the community-summary index for global retrieval and integration. LLM-Only (DeepSeek-R1): no local knowledge base is used, to assess domain coverage and hallucination suppression.

We use Qwen3-30B as the base generation model within Crop GraphRAG. For ablations, we compare against Generic RAG, GraphRAG-wo-Community, and GraphRAG-wo-KnowledgeGraph. For broader baselines, we include DeepSeek-R1, BM25+LLM, and Naive RAG.

### 3.3 Evaluation metrics and methods

The reference answers used for evaluation in this study were not generated by models. Instead, annotators compiled them from authoritative sources (research articles, national and provincial prevention and control guidelines, key information from pesticide registrations and labels, and entries from expert-reviewed professional websites), followed by multiple rounds of verification by domain experts. Given the complexity of agricultural question answering, traditional automatic metrics struggle to capture the true quality of responses. In the ablation experiments, we therefore compute accuracy, recall, and BLEU to enable a comprehensive comparison of our method. The specific evaluation criteria are as follows:

1. Precision. Domain experts in crop diseases and pests evaluate the proportion of correct answers among all system outputs. Let $A_{c}$ denote the number of correct answers returned by the system, and $A_{r}$, the total number of answers returned; the precision percentage is then computed accordingly. This metric provides a direct indication of whether the system’s responses are correct, defined as Equation 19.

(19) $$\text{Precision}=\frac{{\text{A}}_{c}}{{\text{A}}_{r}}$$

2. Recall. Using the reference answers as ground truth, we measure the proportion of correct answers retrieved by the system. Let $A_{s}$ denote the total number of reference answers and $A_{c}$ the number of correct answers returned by the system; the recall value is obtained by computing the ratio $A_{c}$ to $A_{s}$, it is computed as Equation 20.

(20) $$\text{Recall}=\frac{{\text{A}}_{c}}{{\text{A}}_{s}}$$

3. F1 score. The F1 score (Wu et al., 2025) is a key composite metric for classification, information retrieval, and question-answering systems, designed to balance precision and recall. In many practical settings (e.g., agricultural QA), high precision or high recall alone does not reflect overall performance: very high precision with low recall means the system answers only a small subset of queries, whereas high recall with low precision yields many but error-prone answers. Defined as the harmonic mean of precision and recall, the F1 score provides a single summary of both aspects. The calculation is given in Equation 21.

(21) $${\text{F}}_{1}=\frac{2\times \text{Precision}\times \text{Recall}}{\text{Precision}\times \text{Recall}}$$

4. BLEU score. Originally developed for machine translation evaluation, BLEU is now widely used in question answering and other natural language generation tasks. It relies on n-gram matching, and BLEU-4 is commonly adopted to assess the consistency between automatically generated answers and human reference answers (Chauhan et al., 2023). The core formulation consists of modified n-gram precision and a brevity penalty. The specific computation for the n-gram precision term is given in Equation 22.

(22) $$\text{BLEU}=\text{BP}\cdot \exp\left(\sum_{n=1}^{N}w_{n}\cdot \log p_{n}\right)$$

Here, N is the maximum n-gram order; in BLEU-4 we set *N*=4, which balances local lexical matches and short phrasal structure by considering 1-gram, 2-gram, 3-gram, and 4-gram levels. $p_{n}$ denotes the modified precision for n-grams of order n. The weights $w_{n}$are typically uniform; $w_{n}=\frac{1}{N}$, implying equal contribution from each n-gram order, though they may be adjusted for specific tasks. BP is the brevity penalty, where c is the candidate length (the number of tokens in the model’s answer) and r is the reference length (the number of tokens in the human answer); when multiple references are available, r is usually taken as the length of the reference closest to c, as shown in Equation 23.

(23) $$\text{BP}=f\left(x\right)=\{\begin{array}{c} 1\;\;\;\;\;\;\;\;\;\;\;\;,\;if\;c>r\\ \text{exp}\left(1-\frac{r}{c}\right),\;\;if\;c\leq r \end{array}$$

4. Average response time. This metric measures the mean time the system requires to answer a single question. For the 200-question evaluation set, we sum the per-question latencies and divide by the number of questions to obtain the average response time.

In the comparative experiments, we evaluate and contrast systems using the following four criteria:

1. Accuracy: whether the factual information is correct and whether any fabrication is absent.
2. Comprehensiveness: whether the answer covers all key aspects of the question, with particular attention to the completeness of responses to analytical questions.
3. Relevance: whether the generated answer remains closely aligned with the query and avoids irrelevant or redundant content.
4. Traceability: whether the generated answer can be linked back to the supplied contextual evidence.

### 3.4 Question–answer comparison

**Figure 5:**
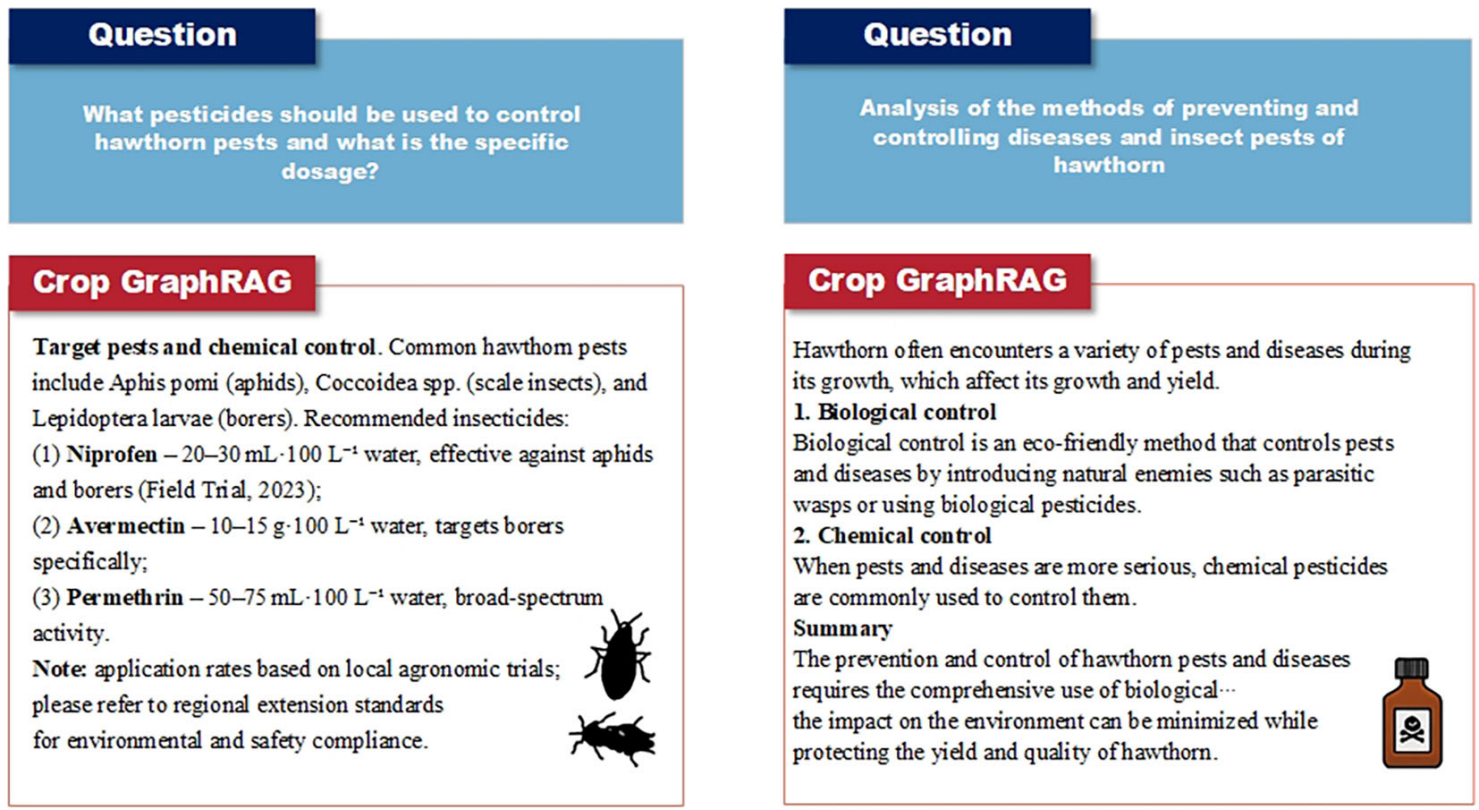
Example responses from Crop GraphRAG to two questions on hawthorn pest and disease management. The left panel presents recommended chemical pesticides and dosages for common pests and diseases; the right panel outlines an integrated pest management strategy that combines biological and chemical control.

We apply the evaluation dataset to the constructed Crop GraphRAG model and conduct comparative question–answering experiments. Representative QA examples are shown in **Figure 5**.

### 3.5 Ablation studies

By sequentially posing the evaluation questions to each model, we obtained detailed answers under the various techniques and conducted comparative analyses. We fed the factual and analytical subsets of the evaluation questions to each method separately, assessed the outputs, and derived the experimental results. Statistical procedures and CLD annotations follow the unified protocol described in 3.6 Statistical analysis. The detailed results are presented in **Table 3**.

**Table 3:** Detailed results of ablation experiments.

Method	Accuracy (%)	Recall (%)	F1 score (%)	BLEU score	Avg response time(s)
GraphRAG-wo-KnowledgeGraph	84.1 ± 0.9^c^	80.5 ± 1.0^c^	82.2 ± 0.8^c^	0.59 ± 0.03^c^	4.1 ± 0.2^b^
GraphRAG-wo-Community	77.2 ± 1.1^b^	75.5 ± 1.4^b^	76.4 ± 1.0^b^	0.48 ± 0.04^b^	3.9 ± 0.2^c^
RAG	72.6 ± 1.3^d^	68.3 ± 1.6^d^	70.3 ± 1.5^d^	0.41 ± 0.04^d^	3.2 ± 0.3^a^
Crop GraphRAG	**89.4 ± 0.8** ^a^	**86.7 ± 0.9** ^a^	**88.0 ± 0.7** ^a^	**0.68 ± 0.03** ^a^	**4.5 ± 0.1^d^**

Different superscript letters within a column indicate significant differences among variants at a = 0.05 after Holm correction; identical letters indicate no significant difference. Bold values indicate the best performance.

**Figure 6:**
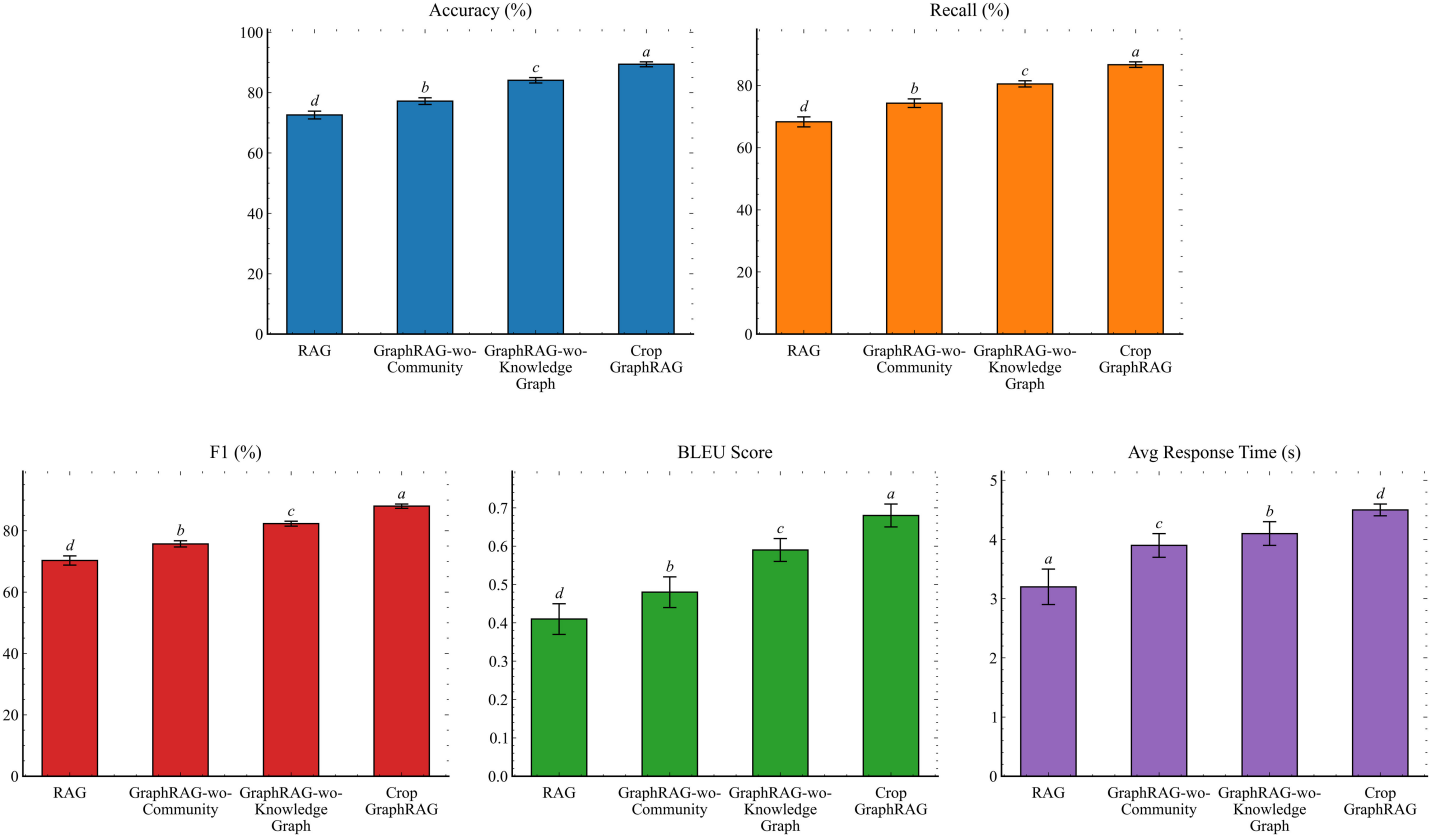
Ablation results on four metrics (Accuracy, Recall, F1, BLEU) and average response time. Error bars denote 95% CIs across runs; superscript letters above bars indicate CLD groups within each metric (α = 0.05, Holm-corrected).

Based on the comparative analysis of the experimental results, **Figure 6** presents the model-wise comparisons.

Taken together, **Table 3** and **Figure 6** show that Crop GraphRAG delivers the best overall quality, with 89.4% accuracy, 86.7% recall, and a BLEU score of 0.68, while maintaining a mean latency of 4.5 s. Removing Community Summaries produces the largest drop: accuracy falls to 77.2%, a decrease of 12.2 percentage points relative to the full model, and BLEU decreases to 0.48. Eliminating the Knowledge Graph also reduces performance to 84.1% accuracy and 80.5% recall, yet it still outperforms naive RAG, which attains 72.6% accuracy, 68.3% recall, and a BLEU score of 0.41. Latency across variants ranges from 3.2 to 4.5 s, indicating an acceptable trade-off between quality and efficiency.

Through comparative experiments, the Crop GraphRAG framework demonstrated superior performance on question answering for crop diseases and pests, particularly for both factual and analytical queries, effectively improving answer quality. The results further confirm that community summaries and the use of a knowledge graph provide strong support for high-quality responses in this specialized agricultural domain.

### 3.6 Comparative experiments

For the comparative experiments, we adopted an LLM-as-judge protocol with human oversight to evaluate our model against several representative baselines (Zheng et al., 2023). To stabilize evaluation without introducing model-identity bias, we anonymized systems as A/B/C/D during review. For each question, absolute scores from 1 to 5 (1 = lowest) were assigned on four dimensions: Accuracy, Coverage, Relevance, and Traceability. All samples were reviewed in three independent rounds with different random orders, and scores were averaged across rounds to obtain the final score. We used ChatGPT-o3 as the judge with a unified prompt template and a fixed context length to avoid systematic bias from context length or template differences. We performed five randomized scoring replications(n=5). Detailed scores and variances for the five rounds of evaluation are shown in **Table 4**.

**Table 4:** Comparative experiment scoring (mean ± SD over five independent evaluation rounds).

Method	Accuracy	Coverage	Relevance	Traceability
Naive RAG	4.00 ± 0.06^b^	4.2 ± 0.05^b^	4.4 ± 0.07^a^	3.8 ± 0.06^b^
BM25+LLM	3.88 ± 0.07^b^	3.61 ± 0.06^c^	4.1 ± 0.05^b^	3.7 ± 0.07^b^
DeepSeek-R1	3.12 ± 0.05^c^	3.55 ± 0.08^c^	4.1 ± 0.06^b^	2.5 ± 0.05^c^
Crop GraphRAG	**4.81 ± 0.04^a^**	**4.30 ± 0.05^a^**	**4.50 ± 0.04^a^**	**4.40 ± 0.05^a^**

Values are mean ± SD (or mean ± 95% CI). Different superscript letters within a column indicate significant differences among methods at a = 0.05 after Holm correction; identical letters indicate no significant difference. Bold values indicate the best performance.

**Figure 7:**
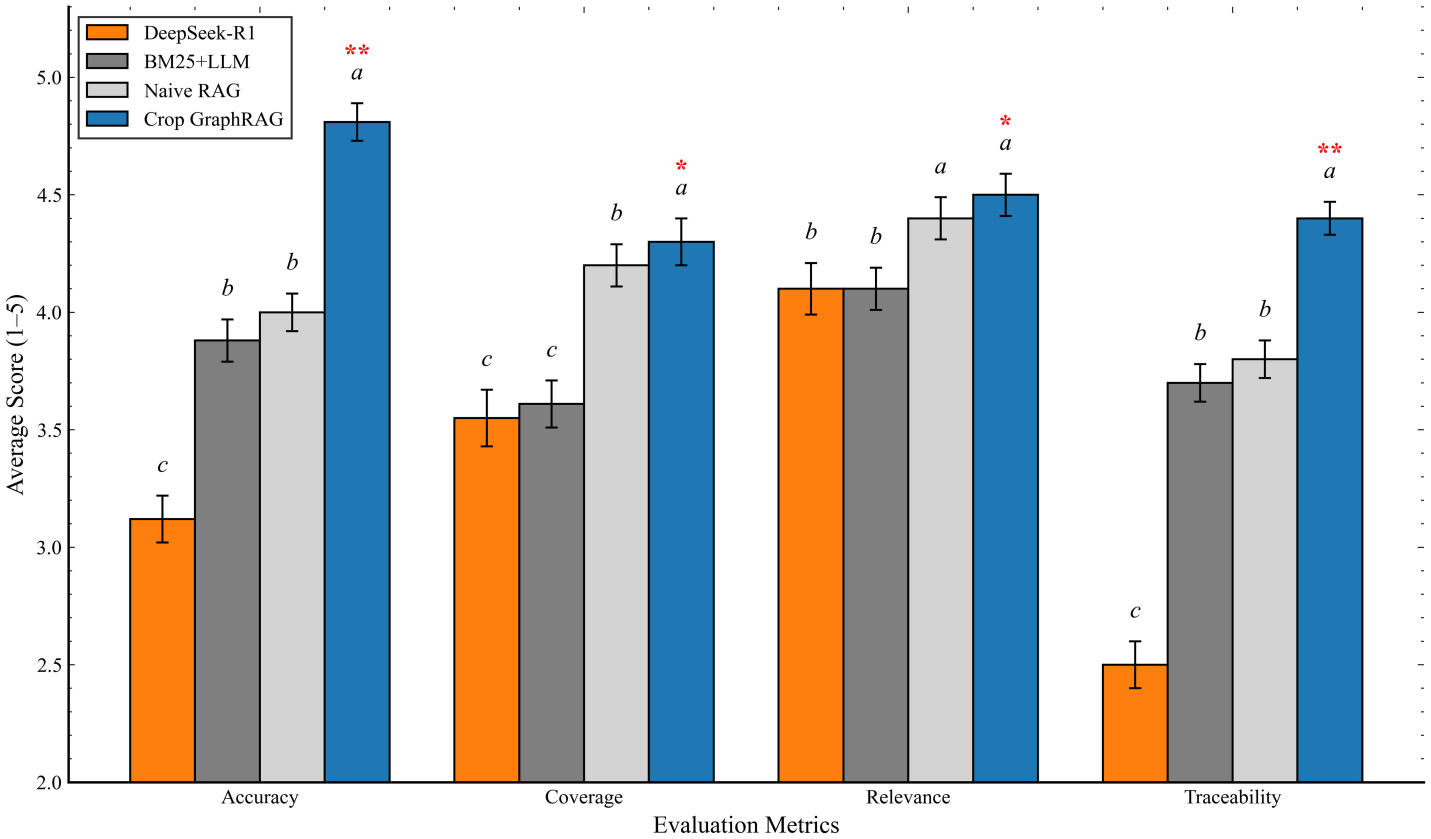
Comparative results with statistical significance (mean ± 95% CI; *p<0.05, **p<0.01). Bars show mean scores; error bars show 95% CI. Red asterisks indicate significance of Crop GraphRAG vs. baselines. Superscript letters denote CLD groups at α = 0.05 after Holm correction.

Statistical analysis. For both the ablation and comparative experiments, we evaluated all methods on the same set of questions repeated across five randomized replications (n = 5), yielding per-question paired observations. We tested whether Crop GraphRAG (or the Full variant in ablations) outperforms each comparator on the four quality dimensions using paired t-tests (Okoye and Hosseini, 2024) and, where normality was questionable, Wilcoxon signed-rank tests (Taheri and Hesamian, 2013). One-sided alternatives were used for quality metrics (H_1_: Crop GraphRAG/Full > comparator) and reversed for latency (H_1_: Crop GraphRAG/Full< comparator). To control multiplicity across pairwise comparisons within each metric, p-values were Holm-adjusted. We then derived compact letter displays (CLDs) from the adjusted pairwise tests so that groups sharing a superscript letter are not significantly different at α = 0.05, while different letters indicate significant differences. Results are reported as mean ± SD and visualized with 95% confidence intervals; CLD letters are annotated in **Table 3** and **Figure 6** (ablation) and in **Table 4** and **Figure 7** (comparative). **Table 5** summarizes the adjusted p-values for the comparative experiment; ablation significance is reflected by the CLD letters in **Table 3**/**Figure 6**. In **Figure 7** we additionally overlay significance asterisks to highlight Crop GraphRAG versus each baseline (* p< 0.05; ** p< 0.01).

**Table 5:** Results of statistical significance tests comparing Crop GraphRAG with the three baseline methods.

Index	vs. BM25+LLM (P value)	vs. Naive RAG (P value)	vs. DeepSeek-R1 (P value)
Accuracy	0.0047	0.0062	<0.001
Coverage	0.012	0.018	0.003
Relevance	0.021	0.031	0.008
Traceability	0.005	0.01	<0.001

Integrated discussion (**Tables 4**, **5**; **Figure 7**). Crop GraphRAG attains the highest mean scores on Accuracy, Coverage, Relevance, and Traceability (**Table 4**), with the largest and most stable gains in Accuracy and Traceability (4.81 ± 0.04 and 4.40 ± 0.05). **Figure 7** shows 95% confidence intervals that are clearly separated in favor of Crop GraphRAG. Pairwise tests in **Table 5** confirm these results: all four dimensions are significant at *α* = 0.05 against every baseline, and several remain highly significant at *p*< 0.01, including Accuracy and Traceability versus DeepSeek-R1 (p<0.001). Overall, combining graph-structured evidence with community-level summaries yields reliable improvements in both correctness and evidence traceability.

## 4 Conclusion

Intelligent diagnosis and control of crop diseases and pests is a key link in safeguarding global food security. The development of intelligent question answering for this domain still faces many challenges, including the sheer volume of unstructured agricultural knowledge and data, and the limited accuracy of general-purpose large language models when applied to specialized agricultural contexts.

This study proposes and implements the Crop GraphRAG framework for the crop disease and pest knowledge domain. The framework converts large volumes of unstructured data into a structured knowledge graph for storage and integrates the generative reasoning capabilities of large language models to build a local knowledge base tailored to this domain. In doing so, it enables efficient knowledge-based retrieval and enhances the question–answering capability of large language models for specialized agricultural tasks. The main findings are as follows:

1. We constructed a domain-specific knowledge graph for crop diseases and pests. From a large corpus of scholarly literature and online resources, we extracted and integrated multidimensional entities encompassing insect pests, diseases, symptoms, and control measures. By leveraging relations among these entities, we organized them into a semantically rich network that serves as a dedicated knowledge base for this domain.
2. We designed and implemented an innovative Crop GraphRAG framework that selects answering strategies according to the user’s query. The retrieval method is chosen dynamically by query type: for factual questions, the system performs entity centric local querying to precisely locate information; for analytical questions, it applies global querying with community summaries to integrate and synthesize the retrieved evidence before answering. These two modes ensure flexibility and applicability of the framework across diverse scenarios.
3. We comprehensively validated the effectiveness of our system through experiments. Results on the evaluation dataset show that the proposed framework clearly outperforms conventional frameworks and retrieval methods across multiple dimensions, including accuracy and comprehensiveness. These findings further demonstrate the strong potential of combining structured knowledge graph storage with large language models for specialized applications in agriculture.

Although the proposed framework demonstrates significant improvements over existing models, certain limitations remain. The response time in information retrieval could be further optimized to enhance real-time performance. Moreover, the current framework focuses mainly on long-text processing, leading to a relatively single information modality. Future research should explore the generalizability of this framework across different domains and languages and incorporate additional modalities—such as visual and sensor data—to enable comprehensive diagnosis and question answering for crop pests and diseases. In addition, modeling and analyzing time-series data, such as emerging pests or disease outbreaks, would allow for dynamic trend prediction. Integrating the framework with real-time agricultural data sources, including meteorological information and IoT-based monitoring systems, could facilitate the construction of a multimodal knowledge graph with both timeliness and adaptability, ultimately advancing intelligent and high-quality agricultural information services.

## Data Availability

The raw data supporting the conclusions of this article will be made available by the authors, without undue reservation.

## References

[B1] Anuar S. H. H. Abas Z. A. Yunos N. M. Zaki N. H. M. Hashim N. A. Mokhtar M. F. . (2021). Comparison between Louvain and Leiden algorithm for network structure: a review. J. Physics: Conf. Ser. 2129, 12028. doi: 10.1088/1742-6596/2129/1/012028

[B2] Arslan M. Ghanem H. Munawar S. Cruz C. (2024). A survey on RAG with LLMs. Proc. Comput. Sci. 246, 3781–3790. doi: 10.1016/j.procs.2024.09.178

[B3] Chandak P. Huang K. Zitnik M. (2023). Building a knowledge graph to enable precision medicine. Sci. Data 10, 67. doi: 10.1038/s41597-023-01960-3, PMID: 36732524 PMC9893183

[B4] Chauhan S. Daniel P. Mishra A. Kumar A. (2023). Adableu: A modified bleu score for morphologically rich languages. IETE J. Res. 69, 5112–5123. doi: 10.1080/03772063.2021.1962745

[B5] Chen C. Li K. Zou X. Li Y . (2021). Dygnn: Algorithm and architecture support of dynamic pruning for graph neural networks. 2021 58th ACM/IEEE Design Automation Conference (DAC). San Francisco, CA, USA. 1201–1206. doi: 10.1109/DAC18074.2021.9586298

[B6] Chen J. Liu Z. Huang X. Wu C. Liu Q. Jiang G. . (2024). When large language models meet personalization: Perspectives of challenges and opportunities. World Wide Web 27, 42. doi: 10.1007/s11280-024-01276-1

[B7] Ding N. Qin Y. Yang G. Wei F. Yang Z. Su Y. . (2023). Parameter-efficient fine-tuning of large-scale pre-trained language models. Nat. Mach. Intell. 5, 220–235. doi: 10.1038/s42256-023-00626-4

[B8] Filipovska E. Mladenovska A. Dobreva J. Kitanovski D. Mitrov G. Lameski P. . (2025). Evaluation of vector databases and LLMs in RAG-based multi-document question answering. ICT Innovations 2024. Communications in Computer and Information Science. (Cham: Springer). 2436, 3–8. doi: 10.1007/978-3-031-86162-8_1

[B9] Harnoune A. Rhanoui M. Mikram M. Yousfi S. Elkaimbillah Z. El Asri B. (2021). BERT based clinical knowledge extraction for biomedical knowledge graph construction and analysis. Comput. Methods Programs Biomed. Update 1, 100042. doi: 10.1016/j.cmpbup.2021.100042

[B10] Jagtap A. D. Shin Y. Kawaguchi K. Karniadakis G. E. (2022). Deep Kronecker neural networks: A general framework for neural networks with adaptive activation functions. Neurocomputing 468, 165–180. doi: 10.1016/j.neucom.2021.10.036

[B11] Jin D. Yu Z. Jiao P. Pan S. He D. Wu J. . (2021). A survey of community detection approaches: From statistical modeling to deep learning. IEEE Trans. Knowledge Data Eng. 35, 1149–1174. doi: 10.1109/TKDE.2021.3104155

[B12] Li M. Yang C. Xu C. Jiang X. Qi Y. Guo J. . (2025). Retrieval, reasoning, re-ranking: A context-enriched framework for knowledge graph completion. Proceedings of the 2025 Conference of the Nations of the Americas Chapter of the Association for Computational Linguistics: Human Language Technologies, Technologies (Volume 1: Long Papers). (Albuquerque, New Mexico: Association for Computational Linguistics). 4349–4363. doi: 10.18653/v1/2025.naacl-long.221

[B13] Liu J. Xing J. Zhou G. Wang J. Sun L. Chen X. (2025). Transfer large models to crop pest recognition—A cross-modal unified framework for parameters efficient fine-tuning. Comput. Electron. Agric. 237, 110661. doi: 10.1016/j.compag.2025.110661

[B14] Liu P. Yuan W. Fu J. Jiang Z. Hayashi H. Neubig G. (2023). Pre-train, prompt, and predict: A systematic survey of prompting methods in natural language processing. ACM computing surveys 55, 1–35. doi: 10.1145/3560815

[B15] McNamara T. P. Chen X. (2022). Bayesian decision theory and navigation. Psychonomic Bull. Rev. 29, 721–752. doi: 10.3758/s13423-021-01988-9, PMID: 34820786

[B16] Mumuni A. Mumuni F. (2025). Automated data processing and feature engineering for deep learning and big data applications: a survey. J. Inf. Intell. 3, 113–153. doi: 10.1016/j.jiixd.2024.01.002

[B17] Ngangmeni J. Rawat D. B. (2025). Swamped with too many articles? GraphRAG makes getting started easy. AI 6, 47. doi: 10.3390/ai6030047

[B18] Okoye K. Hosseini S. (2024). “ T-test statistics in R: Independent samples, paired sample, and one sample T-tests,” in R programming: Statistical data analysis in research ( Springer Nature Singapore, Singapore), 159–186. doi: 10.1007/978-981-97-3385-9_8

[B19] Park M. Tabatabaee Y. Ramavarapu V. Liu B. Pailodi V. K. Ramachandran R. . (2024). Identifying well-connected communities in real-world and synthetic networks. Studies in Computational Intelligence. (Cham: Springer). 1142, 3–14. doi: 10.1007/978-3-031-53499-7_1

[B20] Park M. Tabatabaee Y. Ramavarapu V. Liu B. Pailodi V. K. Ramachandran R. . (2024). Well-connectedness and community detection. PloS Complex Syst. 1, e0000009. doi: 10.1371/journal.pcsy.0000009

[B21] Peng C. Xia F. Naseriparsa M. Osborne F. (2023). Knowledge graphs: Opportunities and challenges. Artif. Intell. Rev. 56, 13071–13102. doi: 10.1007/s10462-023-10465-9, PMID: 37362886 PMC10068207

[B22] Steck H. Ekanadham C. Kallus N . (2024). Is cosine- similarity of embeddings really about similarity? Companion Proceedings of the ACM Web Conference 2024. (New York, NY, USA: Association for Computing Machinery). 887–890. doi: 10.1145/3589335.3651526

[B23] Taheri S. M. Hesamian G. (2013). A generalization of the Wilcoxon signed-rank test and its applications. Stat. Papers 54, 457–470. doi: 10.1007/s00362-012-0443-4

[B24] Upadhyay A. Patel A. Patel A. Chandel N. S. Chakraborty S. K. Bhalekar D. G. (2025). Leveraging AI and ML in Precision Farming for Pest and Disease Management: Benefits, Challenges, and Future Prospects. Ecologically Mediated Development: Promoting Biodiversity Conservation and Food Security. (Singapore: Springer). 41, 511–528. doi: 10.1007/978-981-96-2413-3_23

[B25] Vidya Madhuri E. Rupali J. S. Sharan S. P. Sai Pooja N. Sujatha G. S. Singh D. P. . (2025). Transforming pest management with artificial intelligence technologies: The future of crop protection. J. Crop Health 77, 48. doi: 10.1007/s10343-025-01109-9

[B26] Wan Y. Chen Z. Liu Y. Chen C. Packianather M. (2025). Empowering LLMs by hybrid retrieval-augmented generation for domain-centric Q&A in smart manufacturing. Advanced Eng. Inf. 65, 103212. doi: 10.1016/j.aei.2025.103212

[B27] Wang C. Wang X. Jin Z. Müller C. Pugh T. A. Chen A. . (2022). Occurrence of crop pests and diseases has largely increased in China since 1970. Nat. Food 3, 57–65. doi: 10.1038/s43016-021-00428-0, PMID: 37118481

[B28] Wang L. Chen S. Jiang L. Pan S. Cai R. Yang S. . (2025). Parameter-efficient fine-tuning in large language models: a survey of methodologies. Artif. Intell. Rev. 58, 227. doi: 10.1007/s10462-025-11236-4

[B29] Wang S. Zhuang S. Zuccon G . (2021). Bert-based dense retrievers require interpolation with bm25 for effective passage retrieval. Proceedings of the 2021 ACM SIGIR international conference on theory of information retrieval. (New York, NY, USA: Association for Computing Machinery). 317–324. doi: 10.1145/3471158.3472233

[B31] Wu X. Saraf P. P. Lee G. Latif E. Liu N. Zhai X. (2025). Unveiling scoring processes: Dissecting the differences between llms and human graders in automatic scoring. Technol. Knowledge Learn. 1–16. doi: 10.1007/s10758-025-09836-8

[B30] Wu Q. Zeng J. Wu K. (2022). Research and application of crop pest monitoring and early warning technology in China. Front. Agric. Sci. Eng. 9, 19–36. doi: 10.15302/J-FASE-2021411

[B32] Yan R. An P. Meng X. Li Y. Li D. Xu F. . (2025). A knowledge graph for crop diseases and pests in China. Sci. Data 12, 222. doi: 10.1038/s41597-025-04492-0, PMID: 39915513 PMC11802884

[B33] Yang J. Yao W. Zhang W. (2021). Keyword search on large graphs: A survey. Data Sci. Eng. 6, 142–162. doi: 10.1007/s41019-021-00154-4

[B34] Yao Y. Duan J. Xu K. Cai Y. Sun Z. Zhang Y. (2024). A survey on large language model (llm) security and privacy: The good, the bad, and the ugly. High-Confidence Computing 4, 100211. doi: 10.1016/j.hcc.2024.100211

[B35] Zhang J. Fei J. Song X. Feng J. (2021). An improved Louvain algorithm for community detection. Math. Problems Eng. 2021, 1485592. doi: 10.1155/2021/1485592

[B36] Zhao W. X. Liu J. Ren R. Wen J. R. (2024). Dense text retrieval based on pretrained language models: A survey. ACM Trans. Inf. Syst. 42, 1–60. doi: 10.1145/3637870

[B37] Zheng L. Chiang W. L. Sheng Y. Zhuang S. Wu Z. Zhuang Y. . (2023). Judging llm-as-a-judge with mt-bench and chatbot arena. Adv. Neural Inf. Process. Syst. 36, 46595–46623. doi: 10.48550/arXiv.2306.05685

